# Mental disorders and you: An illustrated and easy guide to mental disorders for the mentally ill and their families

**Published:** 2008

**Authors:** Jacob K. John

**Affiliations:** Department of Psychiatry, CMC, Vellore, India

This refreshingly different volume is interesting from the moment you open the book. It is truly illustrated and has an easy readability throughout. This book is aimed at patients and relatives, but should find itself on the shelf of any mental health professional, particularly the young ones who are fresh out of training and bristling with a theoretical construct of practice. It lays out simple practical management strategies, academically sound but obviously mediated by years of practical down-to-earth experience.

“Mental disorders and you” begins with an introduction which combines its purpose, aims, and the evolution of the book with a large volume of information on the burden of mental illness, statistics, and general associated factors. The next two chapters expand on the aims and a way to use this unusual book. That is well worth a read because it is not really a textbook or in a format like most other textbooks. The reader is taken through page after page of practical questions as raised by patients and their families.

The authors have then arranged information in the traditional way we classify and understand modern psychiatry. Mood disorders, schizophrenia, organic mental disorders, addictions, neurotic, stress related, eating disorders, and problems in development and child psychiatry including mental retardation have all been taken up in detail. The authors have also a chapter on personality disorders and behavior and on different forms of treatments offered in psychiatry.

All these continue in the same informal style of a senior teacher patiently guiding a student through an understanding or a distraught relative through difficult decision making and understanding of their ill relative. It is possibly a good opportunity to look at the authors at this juncture. Drs. Arun and Mary Rukadikar are senior and experienced psychiatrists, who began their journey with mental health in Vellore and went on to complete their training in National Institute of Mental Health and Neuro Sciences. They worked on the faculty at the Wanless Hospital, Miraj before starting their own setup. This background and long years of experience are evident in the writing. Not only in psychiatry, but also in associated medicine as well - how else would a psychiatry text have cute cartoons of “pink puffers” and “blue bloaters.” The authors have commendably stressed on the interfaces of psychiatry with real-time issues in their dealing with gender issues, legal issues, and other aspects of health rather than disease alone.

The book truly reflects the many years of clinical experience and practical questions that patients and relatives normally throw at mental health professionals. The matter being so diverse and unusual, there is a tendency for the reader to feel as though the matter in the book it is slightly disorganized and not easily classified. This negative feeling is enhanced by the large parts of the text being in bold and underlined. This is distracting. For example, a concern like psychosocial rehabilitation of someone chronically ill is distributed in more than eight places in the book without helping the reader tie things together.

These small negatives apart, the book is extremely novel, useful, and a must for all psychiatrists, psychologists, social workers, nurses, and occupational therapists who plan to take up mental health care. Of course, the book is intended for the layman and particularly the patient and the family. There is no doubt that this will fill a great need in that respect. Translation into local languages would be necessary, but worthwhile. It will indeed be a tribute to the authors if they allow the book to be translated into different languages to benefit many more people.

